# Supplementary material for: A stealth adhesion factor contributes to Vibrio vulnificus pathogenicity: Flp pili play roles in host invasion, survival in the blood stream and resistance to complement activation
Source: PLoS Pathog. 2019 Aug 22;15(8):e1007767. doi: 10.1371/journal.ppat.1007767 (PMC6748444; doi:10.1371/journal.ppat.1007767)
Supplement: S1 Fig — (PPTX) [file ppat.1007767.s001.pptx]

## Slide 1
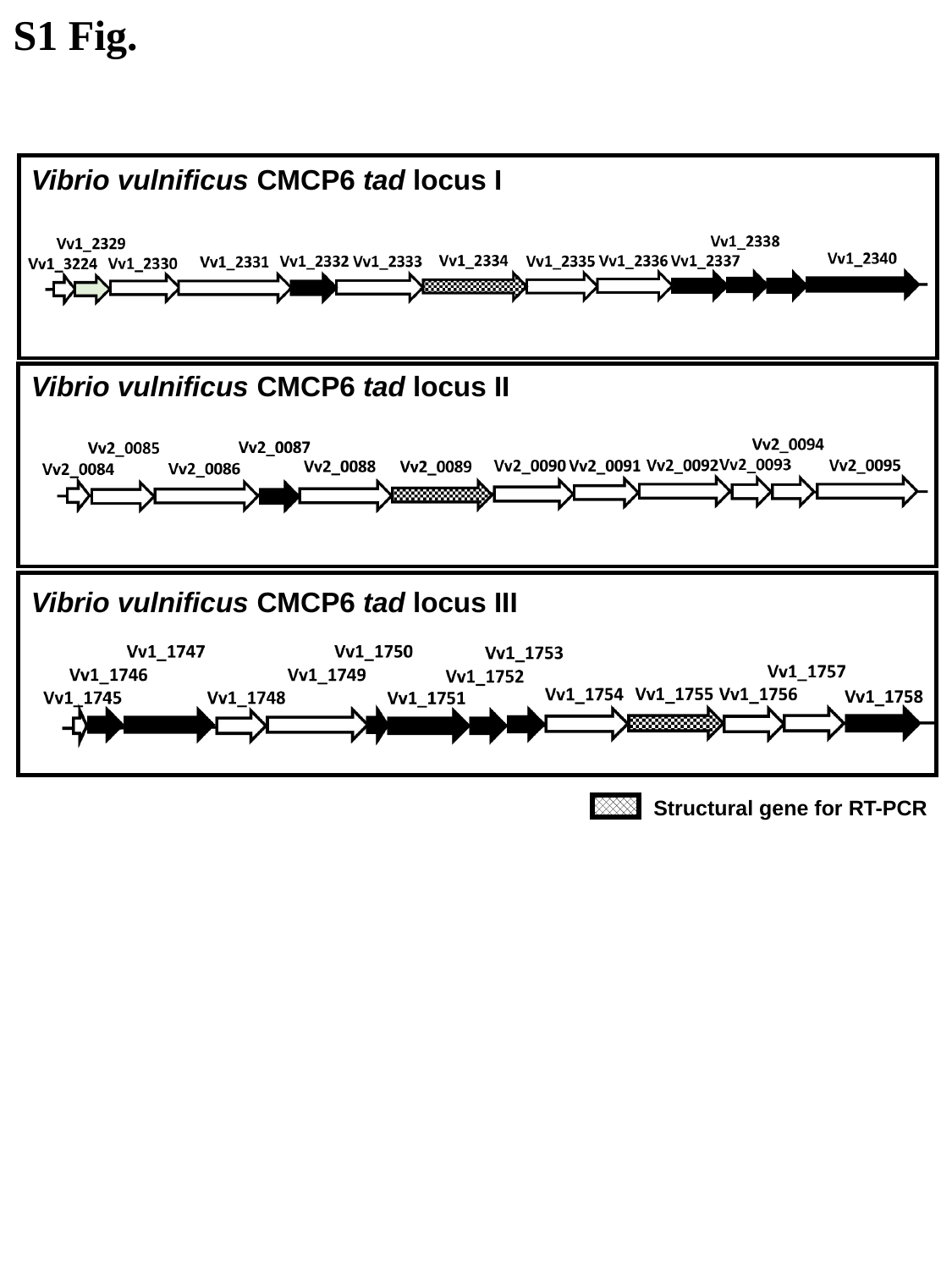

S1 Fig.
Vibrio vulnificus CMCP6 tad locus I
Vibrio vulnificus CMCP6 tad locus II
Vibrio vulnificus CMCP6 tad locus III
Structural gene for RT-PCR
