# Supplementary figures and images for: A stealth adhesion factor contributes to Vibrio vulnificus pathogenicity: Flp pili play roles in host invasion, survival in the blood stream and resistance to complement activation
Source: PLoS Pathog. 2019 Aug 22;15(8):e1007767. doi: 10.1371/journal.ppat.1007767 (PMC6748444; doi:10.1371/journal.ppat.1007767)

## Slide 1
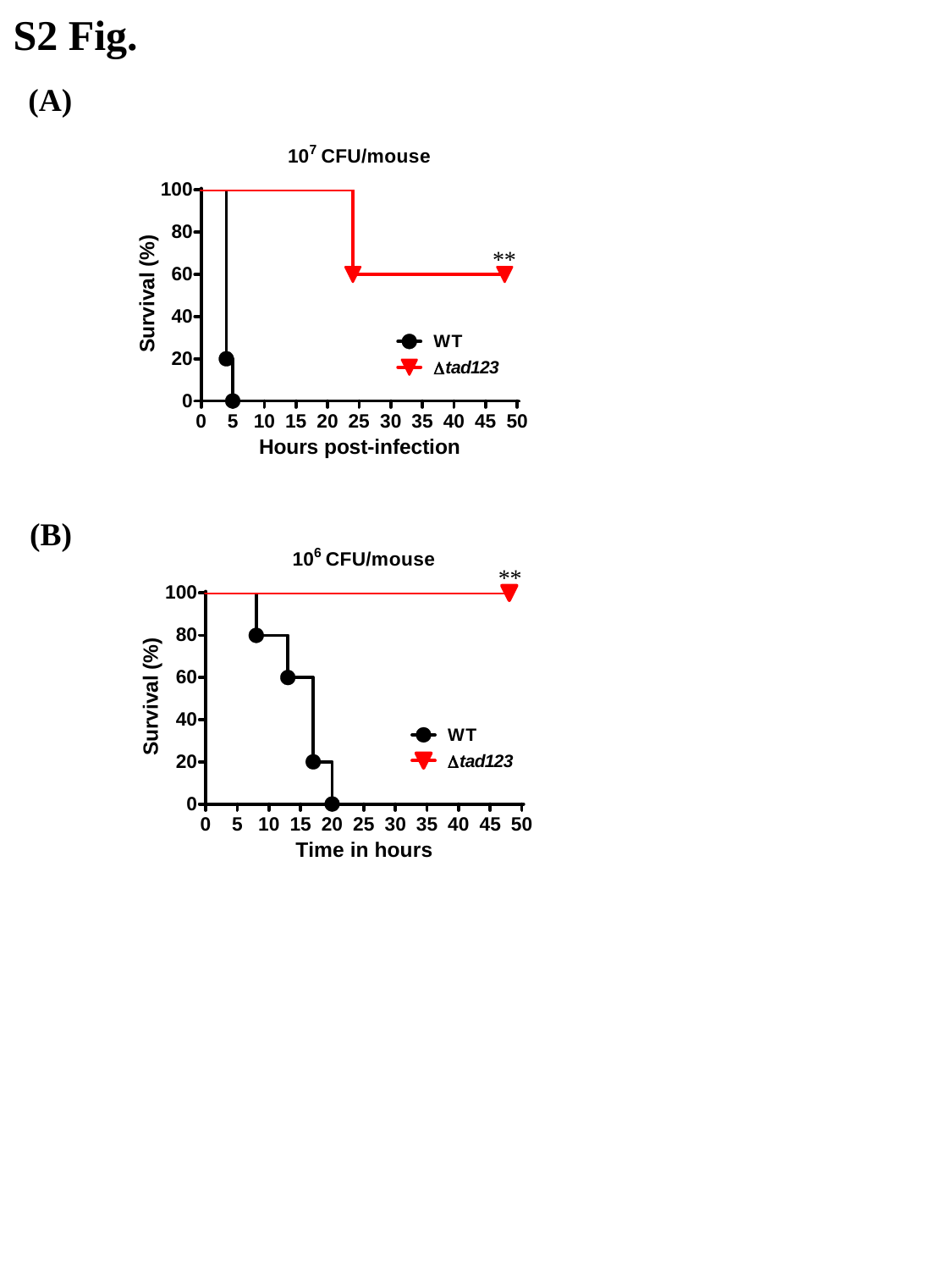

S2 Fig.
(A)
(B)

Supplement: S2 Fig — Seven-week-old randomly bred SPF female ICR mice were intraperitoneally infected with 1 x10^7^ CFU/mouse (A) or 1 x10^6^ CFU/mouse (B) of fresh bacterial suspensions. The challenged mice were monitored for 48 h. Statistical analysis was carried out using Kaplan-Meier analysis followed by the log-rank test (**, P < 0.01). (PPTX) [file ppat.1007767.s002.pptx]

## Slide 1
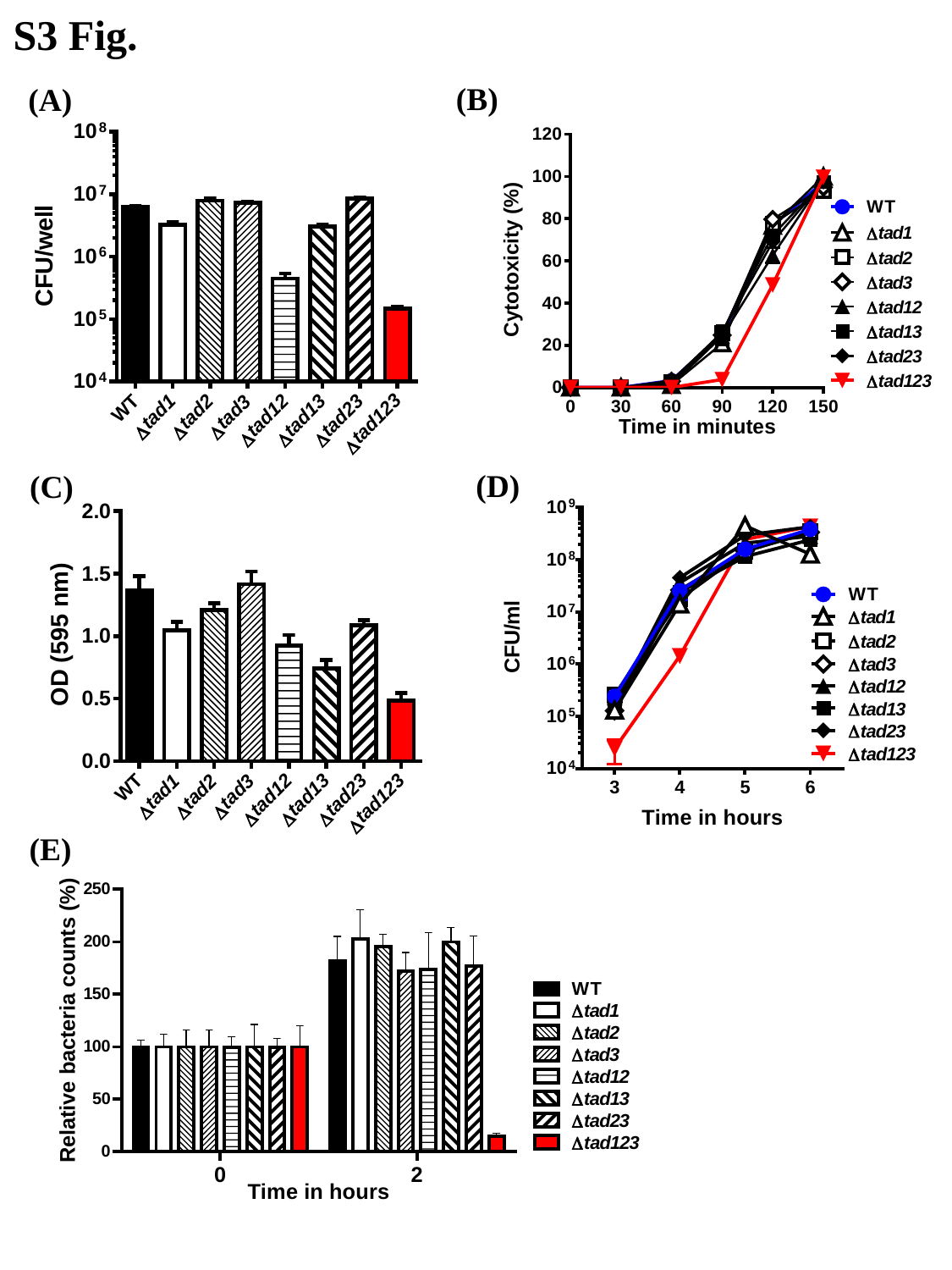

S3 Fig.
(B)
(A)
(D)
(C)
(E)

Supplement: S3 Fig — Log-phase bacteria were used for the experiments. (PPTX) [file ppat.1007767.s003.pptx]

## Slide 1
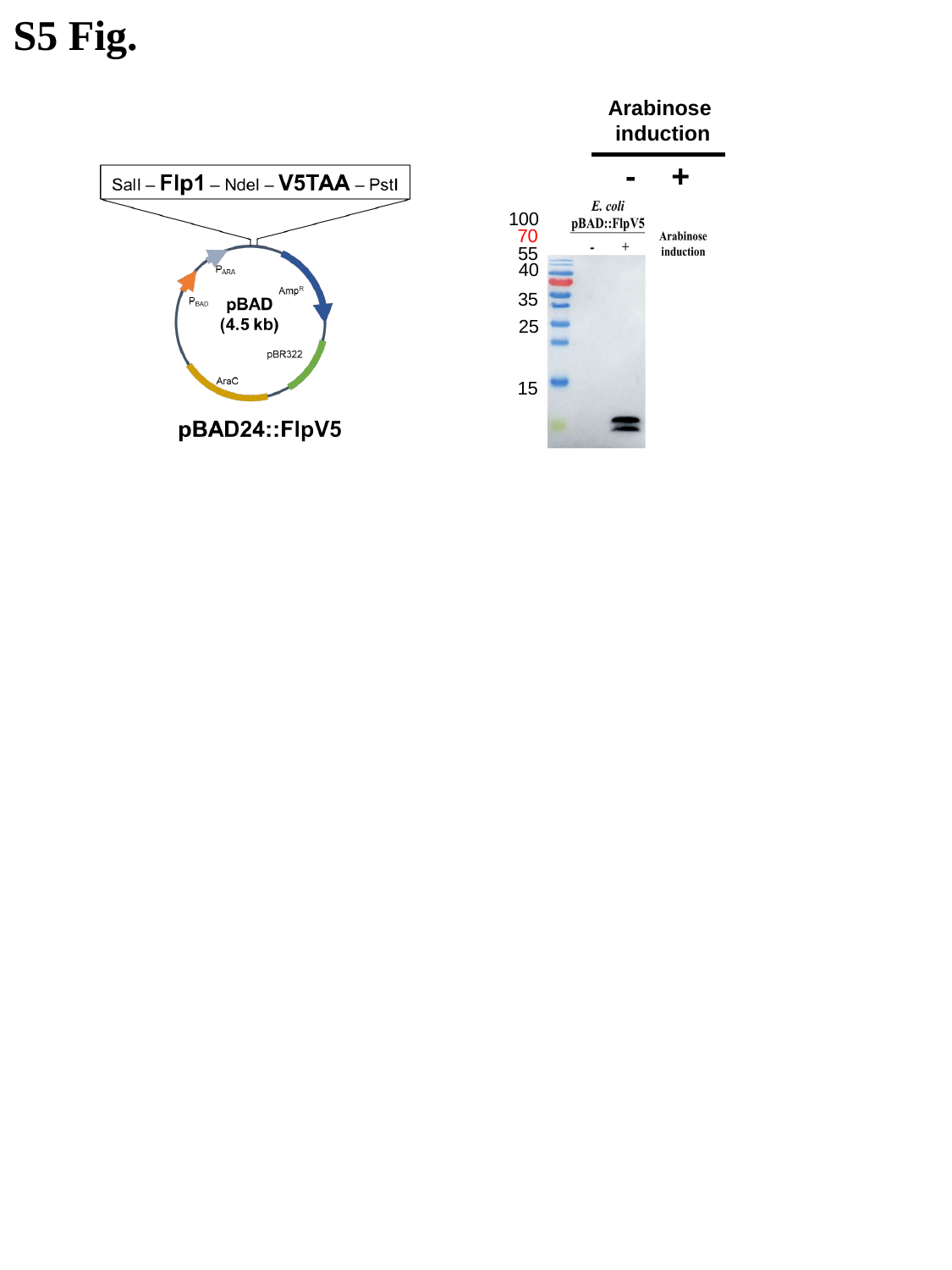

S5 Fig.
Arabinose
induction
- +
100
70
55
40
35
25
15

Supplement: S5 Fig — Bacteria were grown in LB Amp broth supplemented with (inducing) or without (non-inducing) 0.1% arabinose for 4 h. The Flp-V5 fusion proteins were detected using an anti-V5 polyclonal antibody. (PPTX) [file ppat.1007767.s005.pptx]

## Slide 1
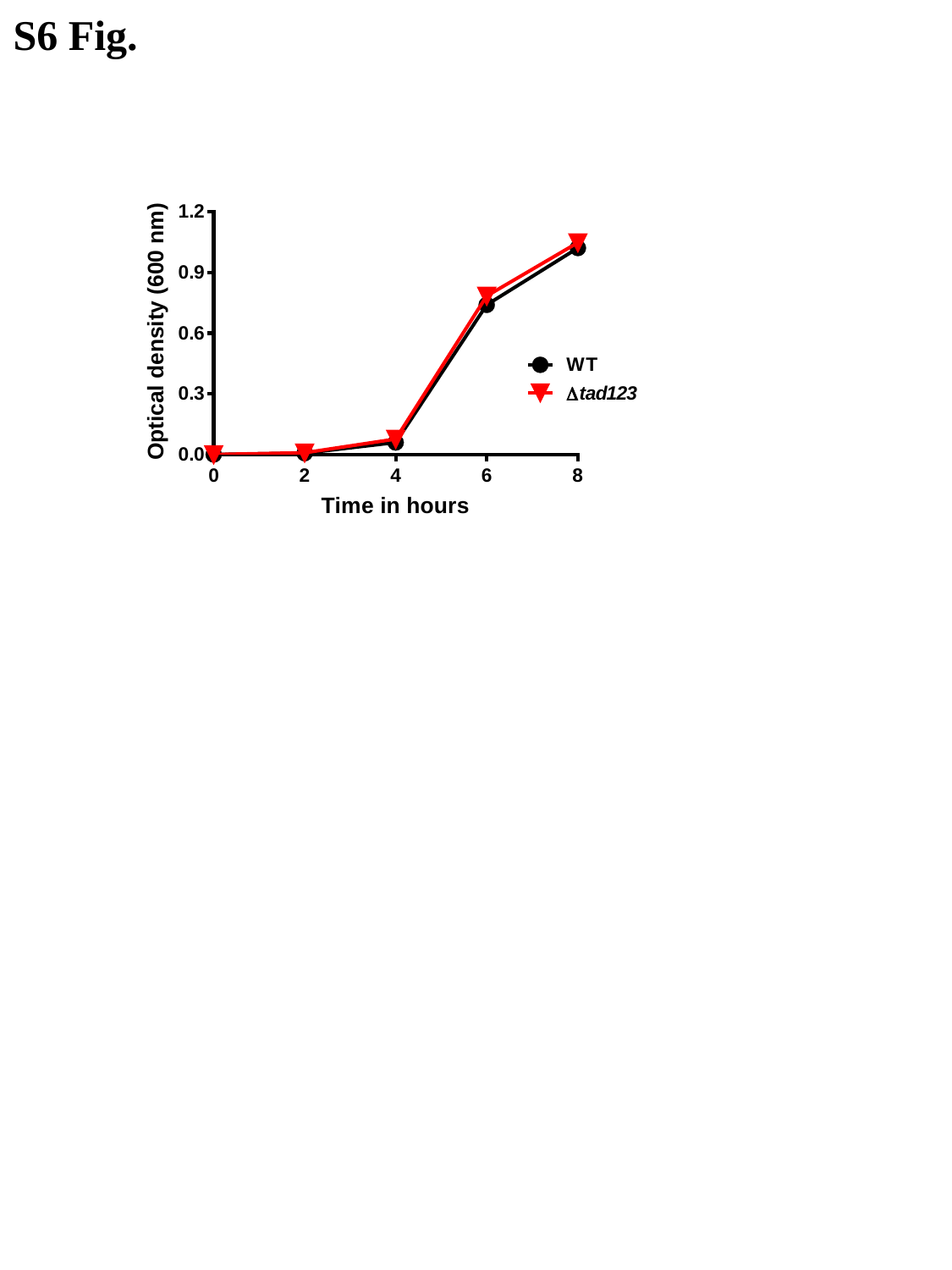

S6 Fig.

Supplement: S6 Fig — Log-phase V. vulnificus cells were grown in high-glucose DMEM, and the OD600 was measured every two hours for 8 h. The growth pattern of the Δtad123 mutant cells was identical to that of the wild-type strain. Data shown represent the mean ± SEM of three independent experiments performed in triplicate. (PPTX) [file ppat.1007767.s006.pptx]

## Slide 1
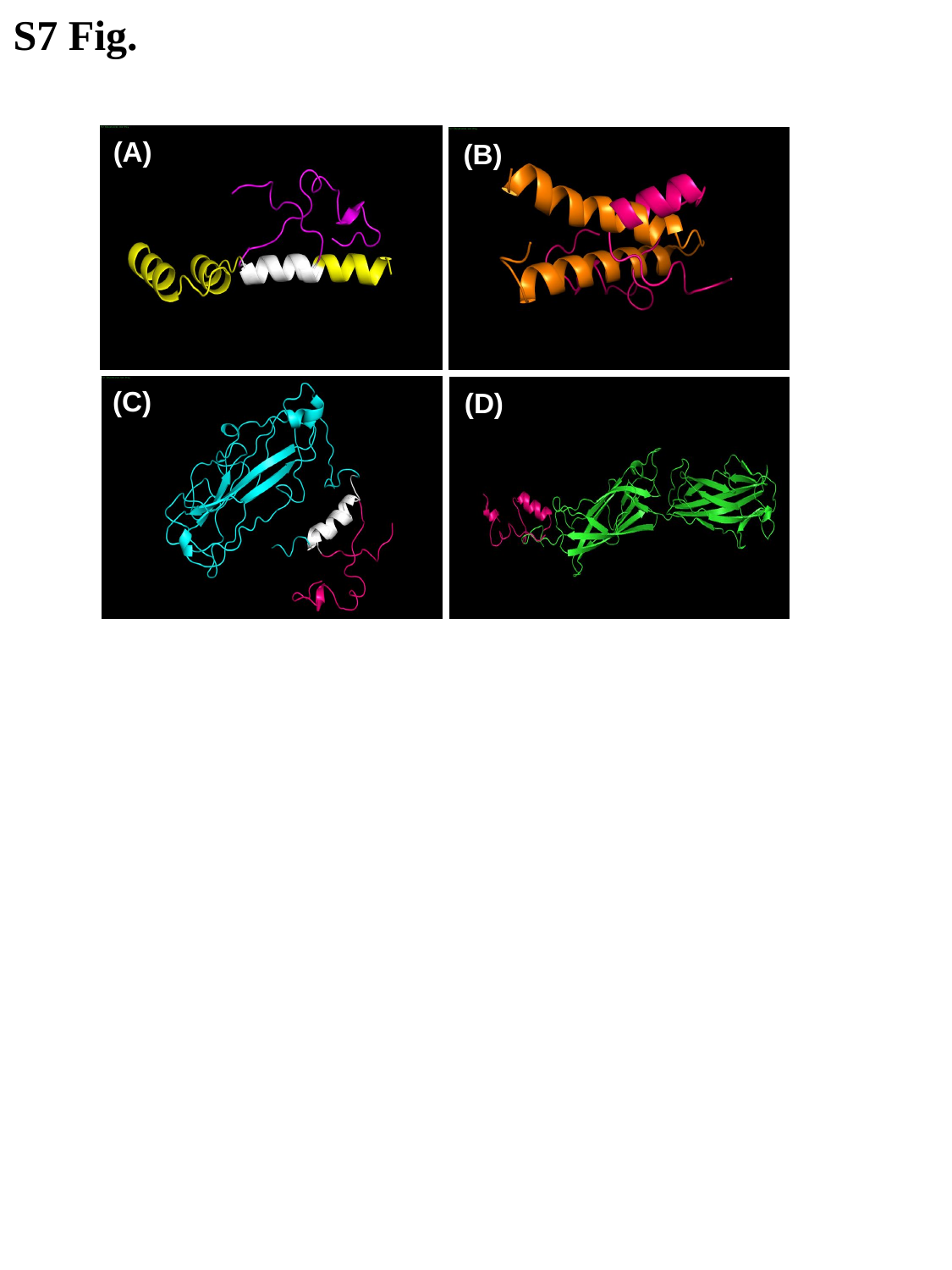

S7 Fig.
(A)
(B)
(C)
(D)

Supplement: S7 Fig — The predicted 3D structure of V. vulnificus Tad pilin (hot pink) was overlaid with those of A. actinomycetemcomitans Flp1 (A, yellow) and Flp2 (B, orange), B. pertussis Fim2 (C, cyan), and E. coli CfaB (D, green). The structures were simulated with the Protein Homology/analogY Recognition Engine V 2.0 (http://www.sbg.bio.ic.ac.uk/phyre2/html/page.cgi?id=index) and superimposed using the MacPyMol version 1.7.4 Education License. (PPTX) [file ppat.1007767.s007.pptx]

## Slide 1
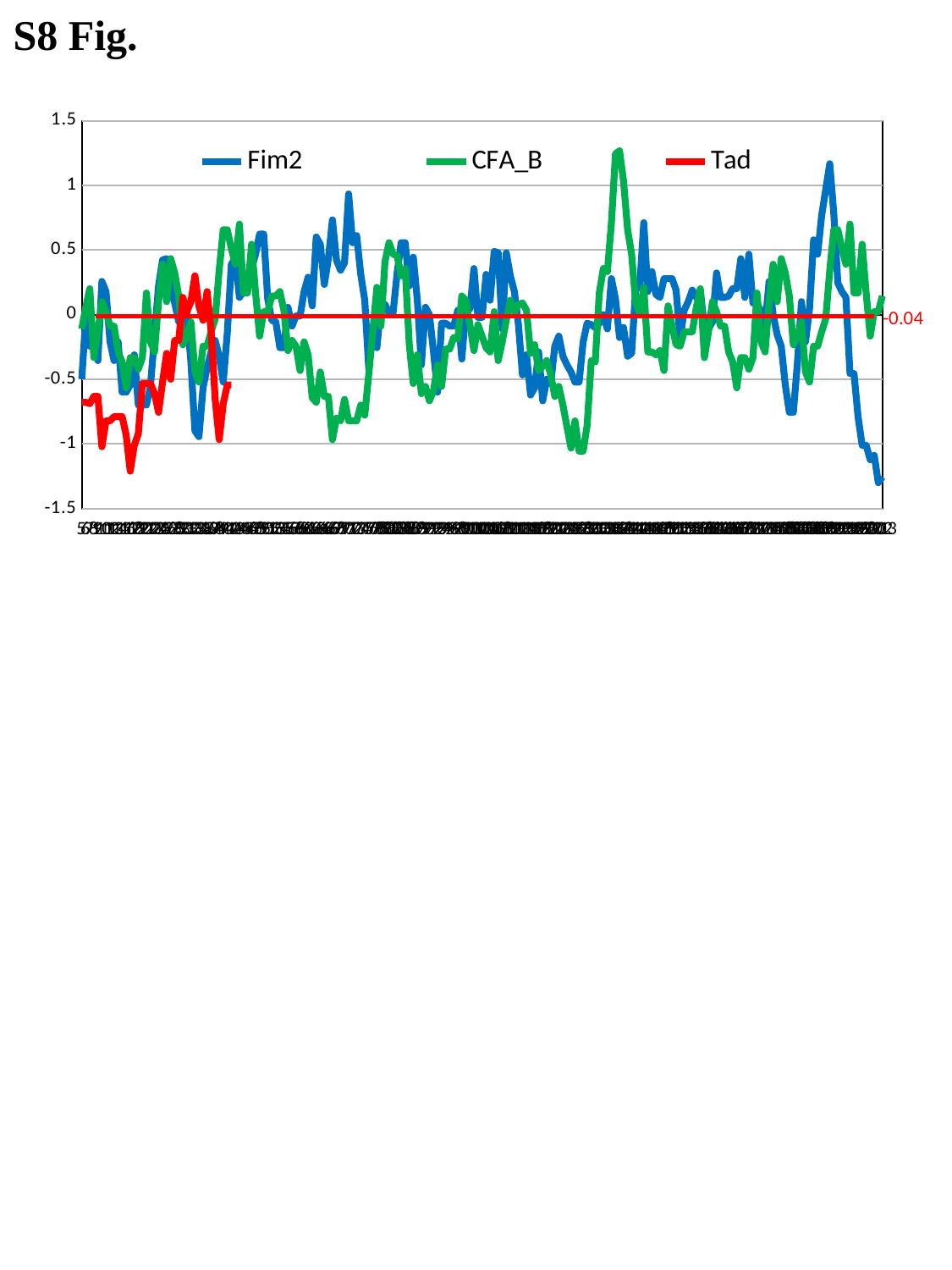

S8 Fig.
[unsupported chart]
-0.04

Supplement: S8 Fig — Positive values indicate hydrophilicity while negative values indicate hydrophobicity. The red line shows the average (Avg) hydrophilicity scores of the Fim2 and CfaB antigenic domains, calculated value of which was -0.04. Only a minor fraction adjacent to the alpha helical region of Tad pilin showed positive hydrophilicity but remained lower than 0.5 hydrophilicity. (PPTX) [file ppat.1007767.s008.pptx]
