# Supplementary material for: A stealth adhesion factor contributes to Vibrio vulnificus pathogenicity: Flp pili play roles in host invasion, survival in the blood stream and resistance to complement activation
Source: PLoS Pathog. 2019 Aug 22;15(8):e1007767. doi: 10.1371/journal.ppat.1007767 (PMC6748444; doi:10.1371/journal.ppat.1007767)
Supplement: S4 Fig — (A) Expression of the structural flp genes was assessed using conventional RT-PCR. RNA was isolated from log-phase bacteria grown in 2.5% NaCl HI broth and then converted into cDNA. RT-PCR was performed using primers specific for each structural flp gene as shown in S2 Table. The 16S rRNA housekeeping gene was employed as the internal control. (B) Bacterial growth in 2.5 HI broth. (PPTX) [file ppat.1007767.s004.pptx]

## Slide 1
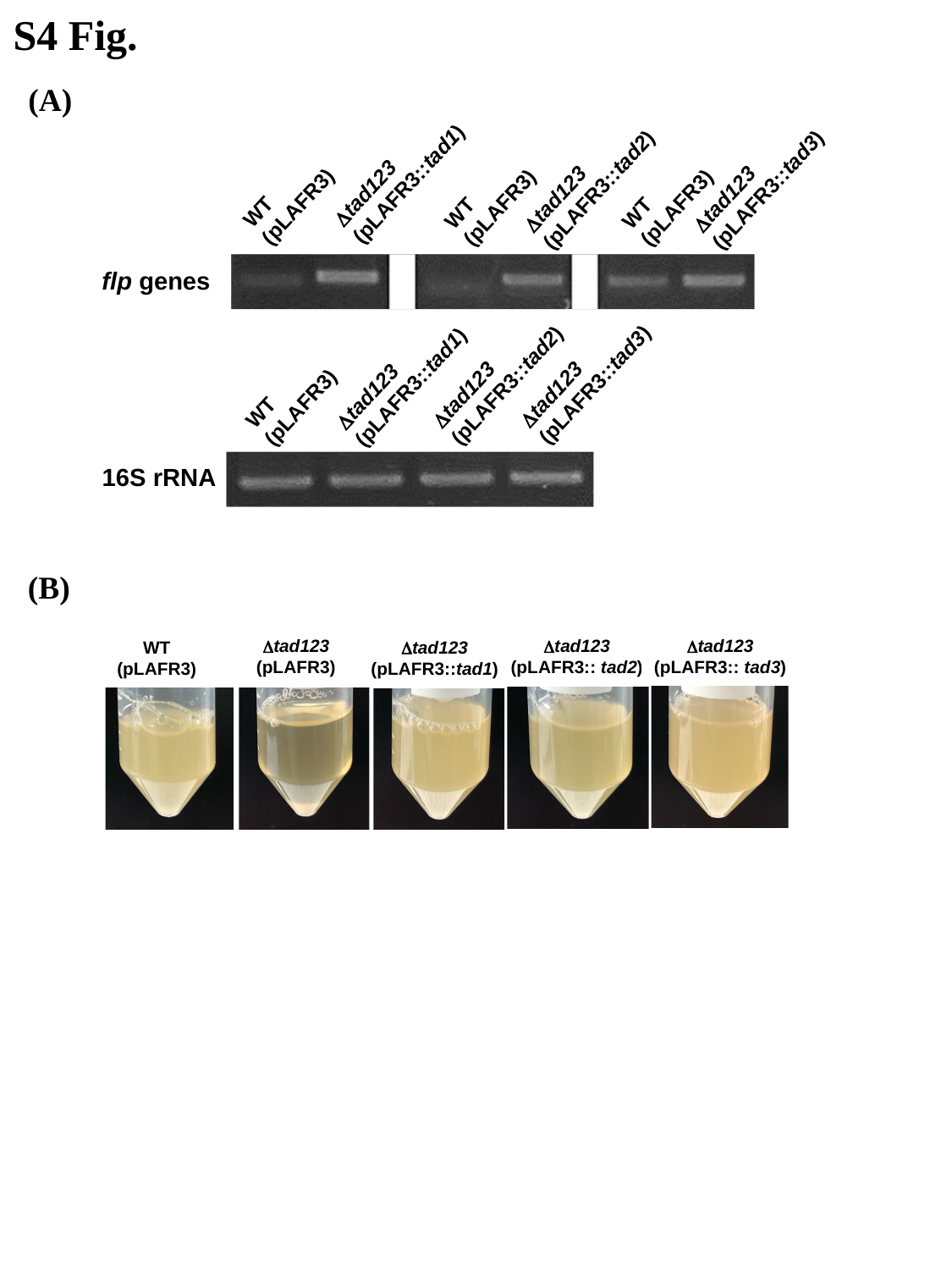

S4 Fig.
(A)
Dtad123
(pLAFR3::tad1)
Dtad123
(pLAFR3::tad2)
Dtad123
(pLAFR3::tad3)
WT
(pLAFR3)
WT
(pLAFR3)
WT
(pLAFR3)
flp genes
Dtad123
(pLAFR3::tad3)
Dtad123
(pLAFR3::tad2)
Dtad123
(pLAFR3::tad1)
WT
(pLAFR3)
16S rRNA
(B)
Dtad123
(pLAFR3)
Dtad123
(pLAFR3:: tad2)
Dtad123
(pLAFR3:: tad3)
Dtad123
(pLAFR3::tad1)
WT
(pLAFR3)
