# Supplementary material for: A stealth adhesion factor contributes to Vibrio vulnificus pathogenicity: Flp pili play roles in host invasion, survival in the blood stream and resistance to complement activation
Source: PLoS Pathog. 2019 Aug 22;15(8):e1007767. doi: 10.1371/journal.ppat.1007767 (PMC6748444; doi:10.1371/journal.ppat.1007767)
Supplement: S1 Table — (DOCX) [file ppat.1007767.s009.docx]

**S1 Table. Primers used for the construction of *tad* operon deletion mutants and the fusion protein.**

| Purpose and primer | Nucleotide sequence (5 to 3) |
| --- | --- |
| *tad1* mutant  tad1-UF  tad1-UR  tad1-DF  tad1-DR | TCCCCCGGGTAAGTATCGCTCACTCTATC  TCTACCCTTGCGTTACAAATGATAACACTTATATTA  AAGTGTTATCATTTGTAACGCAAGGGTAGAAGCCTG  CCGCTCGAGCTGAAAGCCTTTCAATGGCAG |
| *tad2* mutant  tad2-UF  tad2-UR  tad2-DF  tad2-DR | TCCCCCGGGGTAATTAGTCACACAGCATG  TTCATTTTAATCCCATACTATCCTCTTATTAACTT  GAGGATAGTATGGGATTAAAATGAAAAAAACACTG  CCGCTCGAGTTGAGCAAATGAACACTAAC |
| *tad3* mutant  tad3-UF  tad3-UR  tad3-DF  tad3-DR | TCCCCCGGGAGCTCTACATACCATCGTAG  CACATCATCTTGCTACATTAGCTGACTGCCGCGTT  GGCAGTCAGCTAATGTAGCAAGATGATGTGAGGTT  CCGCTCGAGGTACACGGCTTAAAACTGCTC |
| V5-tagged Pilin  Pilin-F  Pilin-R | GCGTCGACTTGCAAATGATATTGATTCTC  CGCCATATGCTTAATCCGGCTGGTGAATAA |

*^a^* Underlined sequences indicate restriction enzyme sites for cloning.
