# Supplementary material for: A stealth adhesion factor contributes to Vibrio vulnificus pathogenicity: Flp pili play roles in host invasion, survival in the blood stream and resistance to complement activation
Source: PLoS Pathog. 2019 Aug 22;15(8):e1007767. doi: 10.1371/journal.ppat.1007767 (PMC6748444; doi:10.1371/journal.ppat.1007767)
Supplement: S2 Table — (DOCX) [file ppat.1007767.s010.docx]

**S2 Table. Primers used in the RT-PCR study**

| Gene | Primer | Nucleotide sequence (5 to 3) |
| --- | --- | --- |
| *flp-1* | flp1-F  flp1-R | GCAAATGATATTGATTCTCATGTC  TTGAGTGCAGAAATAAACACC |
| *flp-2* | flp2-F  flp2-R | AAGTTATGACAAAAGCGATTGA  GAAACACTATCCATCGCATCA |
| *flp-3* | flp3-F  flp3-R | ATGTCGATCTATGAGCGAGTC  TAGCTAAGAGCACCATCGATC |
| *gyrA* | gyrA-F  gyrA-R | GCAGGTGTTCGTGGTATGAAAC  CAACAACACTACCGTTACGCTC |
| *16S rRNA* | 16S rRNA-F  16S rRNA-R | GTTGTGAGGAAGGTGGTGTC  CCGGGCTTTCACATCTGAC |

*^a^* Underlined sequences indicate restriction enzyme sites for cloning.
